# Topical estrogen cream for chondrodermatitis nodularis helicis

**DOI:** 10.1016/j.jdcr.2026.05.044

**Published:** 2026-05-23

**Authors:** Riley Shin, Justin Raman, F. Andrew Pirtle, Michelle B. Tarbox

**Affiliations:** a School of Medicine, Texas Tech University Health Sciences Center, Lubbock, Texas; b Department of Dermatology, Texas Tech University Health Sciences Center, Lubbock, Texas

**Keywords:** chondrodermatitis nodularis chronica helicis, topical estrogen

## Introduction

Chondrodermatitis nodularis chronica helicis (CNCH) is a benign yet often painful inflammatory condition of the auricular cartilage, most commonly affecting the helix and, less frequently, the antihelix in older adults. Clinically, it presents as a tender papule or nodule associated with focal cartilage destruction and overlying epidermal and dermal changes, including erythema and scaling. The primary symptom is localized ear pain, often exacerbated by pressure.^1^, ^2^, ^3^

The pathogenesis of CNCH is not fully understood but is thought to be multifactorial, with chronic mechanical pressure contributing to localized ischemia of the perichondrium and dermis. Histopathologic findings, including epidermal ulceration, dermal inflammation, fibrinoid necrosis, and cartilage degeneration, support an ischemic model of tissue injury. Therefore, therapeutic strategies aim to relieve pressure, reduce inflammation, improve local perfusion, or remove damaged cartilage to restore tissue integrity.^4^

Current management options include pressure-relieving devices, topical or intralesional corticosteroids, nitroglycerin ointment, intralesional hyaluronic acid filler, photodynamic therapy, and surgical excision. Although surgery demonstrates higher reported cure rates compared with nonsurgical therapies, outcomes and recurrence rates vary.^5^^,^^6^

Estrogen has well-established roles in cutaneous physiology, wound healing, and angiogenic regulation. However, its therapeutic use in CNCH has not been previously reported. We report a case of recurrent CNCH resolving with topical estradiol monotherapy refractory to multiple conventional treatments, with reproducible response upon retreatment after recurrence.

## Case presentation

**Fig 1:**
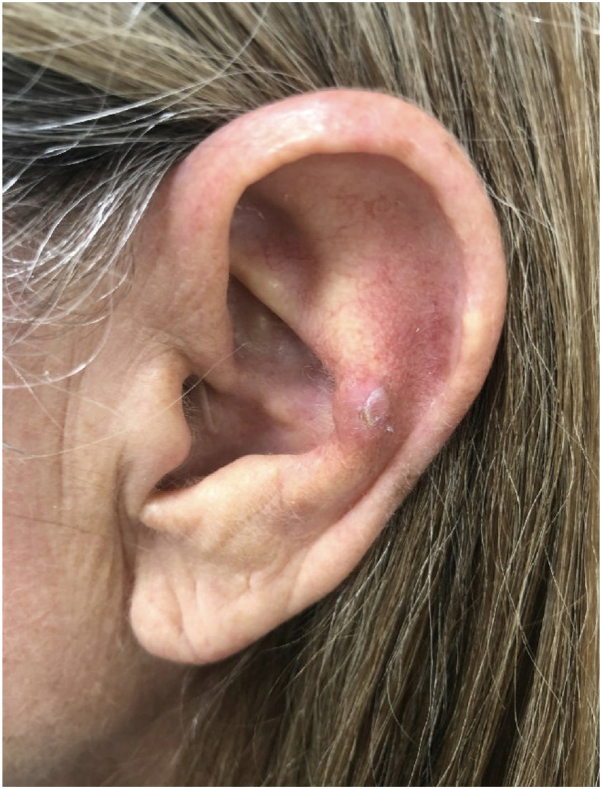
Left antihelix in June 2022 demonstrating a 6 mm erythematous papule with focal scale, consistent with chondrodermatitis nodularis chronica helicis.

A 72-year-old woman with a dermatologic history of nonmelanoma skin cancer presented in March 2021 with a painful lesion of the left antihelix. Initial examination revealed a 6-mm pink papule with focal surface scaling that was tender to pressure, particularly when lying on the affected side. Over the next 3 years, symptoms waxed and waned but never fully resolved despite multiple interventions, including intralesional triamcinolone, high-potency topical corticosteroids (clobetasol 0.05% ointment), pressure off-loading with a donut pillow, hyaluronic acid filler with intralesional corticosteroid, and shave biopsy for lesion removal (Fig 1).

**Fig 2:**
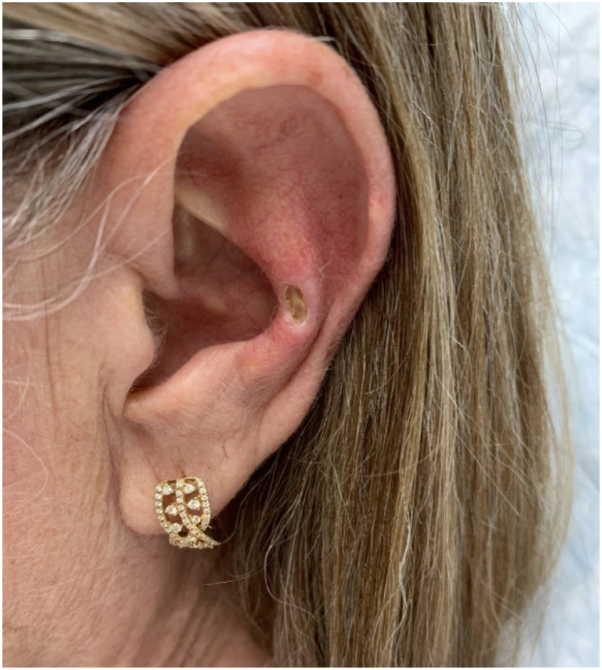
Postshave biopsy appearance of the left antihelix demonstrating a healing surgical site, July 2022.

Shave biopsy performed in June 2022 demonstrated epidermal ulceration with pseudoepitheliomatous hyperplasia, superficial dermal fibrosis, and collagen homogenization, consistent with features of CNCH. Although temporary improvement occurred following procedural treatment, the lesion recurred (Fig 2).

Although clobetasol ointment was continued after biopsy, due to persistent recurrence and ongoing pain, in April 2024, clobetasol was discontinued, and topical estradiol 0.1 mg/g cream was initiated twice daily. Commercially available estradiol 0.01% vaginal cream was used off-label for cutaneous application. Within approximately 1 month, the patient noted reduction in lesion size and tenderness, with complete resolution by December 2024. She subsequently used estradiol intermittently before discontinuing it around August 2025.

**Fig 3:**
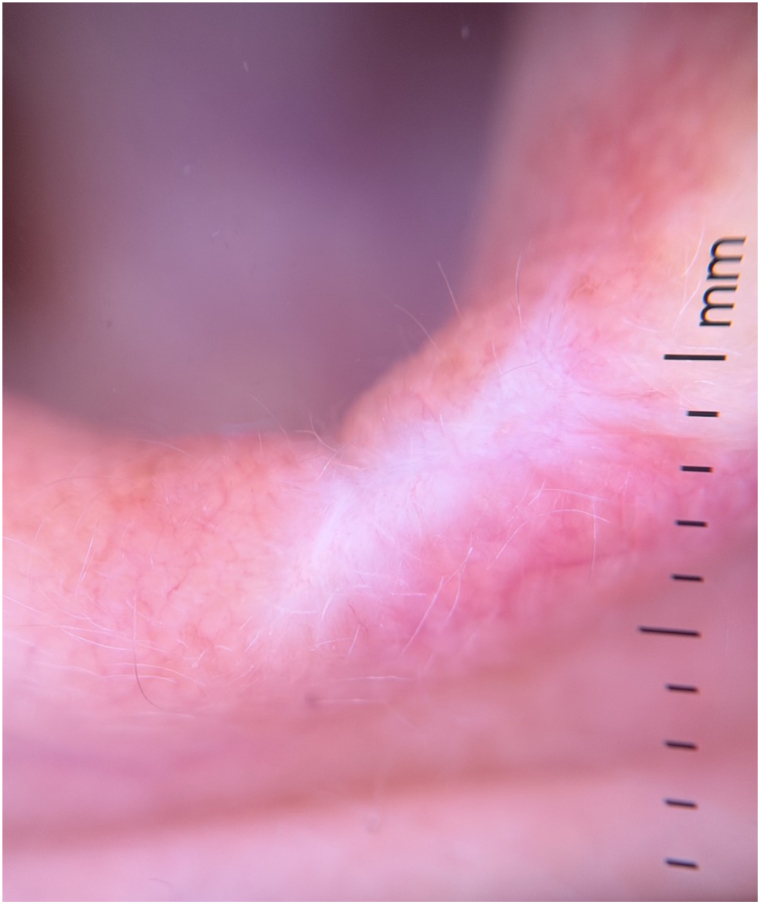
Dermoscopy of the left antihelix in February 2026 demonstrating resolution of the lesion with no residual papule or scaling.

Approximately 5 months after discontinuation, the lesion recurred at the same site in early 2026. She restarted topical estradiol alone and again noted improvement within 1 month. Clinical resolution was noted at follow up in February 2026 on dermoscopy of the asymptomatic lesion (Fig 3). The patient was instructed to continue daily topical estradiol maintenance therapy, and to restart clobetasol daily if the lesion flares.

Of note, the patient had received subcutaneous bioidentical hormone therapy pellets before lesion onset and throughout much of its chronic course. Pellets were discontinued in October 2024. Notably, the lesion persisted during systemic hormone therapy, suggesting a differential effect between systemic and topical estrogen delivery.

## Discussion

CNCH is understood as a localized ischemic disorder of the auricular cartilage and overlying dermis.^1^ The clinical response to pressure off-loading and topical nitroglycerin supports a vascular mechanism. In a retrospective study, topical 2% nitroglycerin produced clinical improvement in over 90% of treated lesions, with complete resolution in more than half.^7^ Nitroglycerin enhances nitric oxide-mediated vasodilation, improving local perfusion and supporting a role for microvascular insufficiency in lesion pathogenesis.

Although surgical excision is associated with higher reported cure rates (∼82%) than conservative measures, recurrence remains possible, and procedural intervention may not be desirable for all patients.^5^ Conservative therapies such as corticosteroids reduce inflammation but do not directly address underlying microvascular compromise and may contribute to dermal thinning with prolonged use. Whereas nitroglycerin provides transient vasodilation, it does not modify structural tissue regeneration.

Estrogen exerts pleiotropic structural and vascular effects in skin. Estrogen signaling occurs through estrogen receptor-α and -β in cutaneous tissue.^8^^,^^9^ Estrogen supplementation improves multiple parameters relevant to tissue resilience and repair, including enhanced matrix production and increased epidermal thickness.^9^ Estrogen deficiency is associated with delayed wound healing, whereas exogenous estrogen use has been shown to accelerate repair in both experimental models and human studies.^8^ Mechanistically, estrogen has been shown to increase transforming growth factor-β associated signaling during repair, promote proangiogenic pathways including vascular endothelial growth factor signaling, and enhance endothelial nitric oxide synthase activity, thereby supporting improved microvascular perfusion.^9^ Given that CNCH can arise from pressure-induced ischemia superimposed on structurally vulnerable skin, estradiol’s ability to enhance perfusion while reinforcing dermal integrity provides a therapeutic rationale.

Systemic versus topical estrogen may be particularly relevant in our case of CNCH. Systemic hormone replacement therapy has been associated with reversal of age-related impairment in cutaneous wound healing in older women.^10^ However, systemic delivery depends on adequate perfusion of the target tissue, and impaired vascular supply can limit drug delivery to poorly perfused wounds.^11^ In CNCH, the affected site is thought to represent a focal pressure-related ischemic lesion with microvascular compromise, which may limit delivery of circulating hormone to the affected site. In contrast, topical application permits direct delivery to the lesion and may increase local tissue exposure despite impaired local blood flow.^11^ Experimental wound-healing literature further supports route-specific differences. In ovariectomized mice, direct topical estradiol benzoate application to wounds reduced inflammation and promoted angiogenesis and wound contraction more than subcutaneous estradiol pellet delivery.^12^

Estrogen-based therapy for CNCH has not previously been reported. This case is strengthened by a reproducible response pattern, with lesion resolution during estradiol use, recurrence after discontinuation, and improvement upon reinitiation. This sequence supports a pharmacologic effect rather than spontaneous remission alone. This report is limited by its single-patient design, and additional cases and studies are needed to evaluate reproducibility and generalizability.

Although causality cannot be established from a single case, the findings presented here suggest that hormonal modulation may warrant further study as a targeted therapeutic strategy in CNCH. Given the recurrent nature of the condition and its impact on quality of life, prospective evaluation of topical estradiol is needed to determine efficacy, optimal dosing, and long-term safety.

## Conclusion

This case describes resolution of recurrent CNCH with topical estradiol monotherapy, demonstrating a reproducible response upon retreatment following recurrence. A literature review did not identify prior reports of estrogen-based therapy for CNCH, suggesting potential novelty. Estrogen’s established effects on angiogenesis, nitric oxide signaling, inflammation, and dermal collagen support provide a possible mechanistic explanation for the observed clinical response. This case highlights a potential alternative therapy for refractory CNCH and raises the possibility that vascular and dermal factors contribute to disease persistence. Further clinical investigation is warranted to evaluate reproducibility and clinical applicability in dermatologic practice.

## Conflicts of interest

None disclosed.
